# Convolutional Neural Network-Based Virtual Reality Real-Time Interactive System Design for Unity3D

**DOI:** 10.1155/2022/2530836

**Published:** 2022-06-22

**Authors:** Hongxia Li

**Affiliations:** School of Arts, Shandong Management University, Jinan 250357, China

## Abstract

In this paper, the algorithm of the convolutional neural network is used for in-depth research and analysis of real-time interactivity, and a virtual reality real-time interactive system is designed based on Unity3D. Aiming at the problems of high computation and low efficiency of existing high-precision models, based on the lightweight network model, this paper proposes a real-time semantic segmentation method based on an asymmetric codec. The encoder part of the network adopts a newly designed bottleneck residual module based on depth-separable convolution, null convolution, and decomposition convolution to extract local and contextual information without increasing the computational effort. At the same time, channel rearrangement is introduced in the module to facilitate the interaction of information between channels. The approach proposed in this paper can present the architectural system simply and clearly and is more flexible to match various complex business relationships. The system design technology is based on the Spring MVC framework technology, using visualization technology design and implementation. The combination of the Spring MVC framework and Unity3D achieves the separation of the front and back ends of the system, which makes the system stable, real-time, visible, and efficient. Meanwhile, a newly designed global attention guidance module based on the attention mechanism is introduced at the jump connection between codecs to guide the low-level features of encoder structure and high-level features of decoder structure for better integration and accuracy improvement.

## 1. Introduction

Environment-aware technology is one of the components of smart car technology, and it is also one of the most important technologies. It uses various sensors to collect information around the vehicle and then sends the information to the information processing terminal, where it analyzes and predicts potential threats around the current vehicle and alerts the driver to drive safely to improve driving safety and reduce accidents [1]. The sensors used in environmental awareness technology generally include cameras, radar, and GPS, among which cameras collect information around the car by taking pictures, and then use convolutional neural networks or traditional image processing algorithms to analyze the pictures to obtain the coordinates and types of cars, pedestrians, and other predefined targets in the pictures and at the same time segment the lane lines or lanes through segmentation algorithms; radar can effectively measure the distance of the target from the vehicle, and the error is smaller compared with the image ranging error, and finally, the car terminal realizes the anticollision function through the distance measured by the radar; while sensors such as GPS can obtain some additional necessary information, such as car position and speed. The target box coordinates; category and preset category mask can be obtained at the same time when the network predicts. Because the multitask deep learning framework adopted in this paper is hard parameter sharing. With the continuous change of different enterprises for enterprise requirements, the level of enterprise architecture system requirements is getting higher and higher, and the application relationship architecture changes more frequently [2]. The actual architecture model after the application architecture transformation may have been dramatically different from the expected, and it is difficult for the architect or system operation and maintenance staff to accurately remember the composition and interaction of all resource instances; secondly, the system architecture may introduce some unreliable factors during the dynamic evolution, such as capacity issues and system performance overcoupling, all of which may add insufficient security performance to the system and system stability may be an impact. So each time we face system transformation, business volume, and stability governance work before, first through the overall system architecture diagram to sort out the specific work requirements, for the latter to present the application relationship between each application component in the application relationship architecture system, application relationship architecture visualization system can help us identify the problems in the overall enterprise architecture while assisting enterprises to establish a high-level, fast easy to use the system.

Current advances in computer technology, supported by GPUs with powerful parallel computing capabilities and high-resolution photographic devices, have led to significant developments in high-precision and high-resolution semantic segmentation. The use of GPUs to accelerate the training of large deep convolutional neural networks (DCNNs) to automatically capture deep features can achieve more accurate semantic segmentation. In recent years, many models have made continuous progress in the accuracy of semantic segmentation, but they generally use hundreds of convolutional layers and thousands of feature channels, which limit the application of semantic segmentation in real-world scenarios such as autonomous driving and virtual reality because they require many resources and cannot be applied to memory as well as computationally constrained mobile devices [3]. Therefore, to be better applicable to mobile hardware devices with cheap deployment and low power consumption, real-time semantic segmentation techniques that can trade-off between accuracy and efficiency of semantic segmentation are also gaining increased attention from researchers [4]. In this paper, we aim to investigate DCNN-based real-time semantic segmentation algorithms to make them perform better on existing street segmentation datasets to provide strong technical support for future industrial implementation.

In recent years, with the development of VR technology, the demand for VR services in various industries is also increasing. Then, in the channel dimension, the output feature maps of the two branches are spliced into one feature map, and finally, the information flows between the channels of the spliced output feature map through the transition layer, and the dimension is reduced and the number of parameters is reduced. This shows that the realization of VR and its application system combined with other industries has important practical significance and economic value. In the development of modern society, the combination of sports and technology has gradually become one of the most active factors in the development of sports. VR applications are limited by the traditional human-computer interaction, the user interaction with the virtual world is generally carried out through locators, handles, and other devices and even many applications require users to wear tracking markers on their bodies, etc., which has a greater impact on some of the higher degree of freedom VR applications. Virtual reality applications combined with motion capture technology can correspond to the real world and the virtual world from a higher dimension to greatly improve the previous VR applications to watch the main situation. To achieve a more immersive and interactive effect, it is necessary to update the motion capture equipment and design new motion capture methods and solutions.

## 2. Related Works

An underwater VR system for diving training is proposed to enable scuba students to experience arbitrary underwater environments and to be able to use VR technology for training in limited water environments. The ImmerTai system was developed to capture the movements of a Tai Chi expert and to be captured in an immersive party CR to perform Chinese Tai Chi exercises [5]. The student's movements are also presented to the PC environment in a multimodal format to deliver the captured movements to the student. Student movements are also captured for quality assessment and used to form a virtual collaborative learning environment. In addition to this, a virtual reality ski training system using an indoor ski simulator is proposed [6]. The system is based on a simple indoor ski simulator with two trackers to capture the motion of the skis. Users can control the skis in the provided virtual ski slopes and can train their skills by replaying professional skiers [7]. The output feature with 3 channels and an image resolution of 512 *∗* 256 is obtained. The United States is the birthplace of cutting-edge virtual simulation technology, and NASA's Ames Laboratory is a major player in the field, making engineering applications of virtual simulation (e.g., data helmets, data gloves, virtual reality simulations of space stations) progressively more sophisticated, and virtual simulation of the Hubble Space Telescope is one of the technological achievements, and NASA has established a related space science virtual simulation training system, as well as multitasking applications in the space station virtual simulation operating system [8].

Because of its tremendous applications in training and testing, virtual reality technology is often used first in sectors such as defense and aerospace and then gradually spread to industries such as healthcare, education, and entertainment, which have developed huge sales markets for it and reaped lucrative economic benefits. In just a few decades, virtual reality technology has become increasingly perfect for every aspect of everyone's daily life, making it possible for managers, leaders, and engineers to make operational use, modification, and design of the designed system in a virtual reality environment to make the system better [9]. Some mature motion capture products and virtual reality interactive solutions do not live up to expectations in terms of experience and are difficult to popularize to general users [10]. Therefore, good motion capture system design and virtual interaction system development for the industry means lower economic costs and a better user experience. As a key issue within the VR field, it has received a lot of attention from researchers [11].

In the microservice architecture visualization system embodying specific applications, a specific analysis is developed for distribution network integration [12]. During the visualization presentation, the application components are simplified, which in turn provides a more meaningful view to enterprise administrators and increases effective management by operations and maintenance staff. Here, meaningfulness and effectiveness are taken as the core starting point, and the application components are effectively displayed, and then, the enterprise managers can clearly identify the application components, identify whether the components in the architecture system are of huge application value, and then find the useful elements associated with them according to the application value of the components and their meaning, and finally visualize the architecture. Secondly, some visualization tools have been used in practice with great results and constantly improve the efficiency of development managers to troubleshoot problems. The number of channels obtained by convolving this output feature with the third 3 *∗* 3 in the encoder is 32, and the image resolution is 512 *∗* 256. Features are spliced. With the upstream and downstream dependency diagrams of the system architecture, developers can quickly locate the source of the problem in case of failure with the help of dependency data, which greatly reduces the problem repair time (MTTR). With the application relationship architecture diagram, it is possible to display the correlation between application components in the system or perform failure simulation for each component that the system depends on to evaluate the reliability of the system in the face of local failures.

## 3. Convolutional Neural Networks for Unity3D Virtual Reality Real-Time Interactive System Analysis

### 3.1. Convolutional Neural Network Algorithm Design

The network can combine high-resolution spatial information and low-resolution depth features. The network first modifies the shortcut connections to learn the downsampling module, including a Conv2D layer with 32 output channels, i.e., a normal convolutional layer, a DWConv layer with 48 output channels, and a 64 layer, i.e., a depth-separable convolutional layer, which ensures that low-level features can be shared and used efficiently and that the convolutional kernel size is 3 *∗* 3. Each layer is followed by a batch normalization layer and an activation layer using the ReLU function.

**Figure 1:**
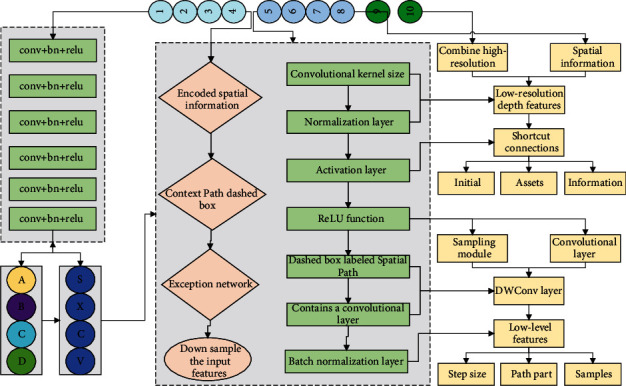
Schematic diagram of BiSeNet network.

The dashed box labeled spatial path in Figure 1 shows the spatial path branch, which consists of three main layers, each of which contains a convolutional layer, a batch normalization layer, and a ReLU layer. Since each convolutional layer uses a normal convolution with a step size of 2, the spatial path part downsamples the original image by a factor of 8, and the resolution of the output features is 1/8 of the original image. Moreover, because of the high resolution of its feature map, the encoded spatial information is richer [13]. The part labeled as the Context Path dashed box in the figure is the context path branch, and its back end is the Exception network, which can downsample the input features quickly to obtain a larger perceptual field and extract advanced semantic information. The 16-fold and 32-fold downsampled features are passed through the Attention Optimization Module (ARM) to obtain features that integrate global contextual information, which is the final output of the context path.

Compared with the traditional deep learning network structure, the hard parameter sharing structure needs to fit multiple tasks during network training, and since each subtask has a certain correlation with each other, each subtask will promote each other during network training, which not only can help the network reduce the risk of overfitting but also can improve the detection accuracy of the network. For example, photos, materials, installation environment, and other pieces of information are used to map, illuminate, render, and bake the model, to finally obtain a virtual reality model with highly realistic visual effects. The soft parameter sharing mechanism is different from the hard parameter sharing mechanism in that each subnetwork has a backbone network, which is equivalent to multiple networks running in parallel.

Although the soft parameter sharing mechanism is a little better than the hard parameter sharing mechanism, the soft parameter sharing mechanism uses a larger number of parameters and a larger amount of computation, and the detection speed is slower. To achieve a real-time detection effect, this paper chooses the hard parameter sharing mechanism, which has a great advantage in detection speed and can save many shared parameters, although it has a simple structure.

In the multitask convolutional neural network, the semantic segmentation feature layer and the target detection feature layer are jointly used as branching feature layers of the multitask convolutional neural network, so that the target frame coordinates, categories, and preset category masks can be obtained simultaneously when the network predicts. Because the multitask deep learning framework used in this paper is hard parameter sharing, the semantic segmentation feature layer and the target detection feature layer share a backbone network and use the same parameters, which can reduce many parameters and can effectively reduce resource consumption.

(1) $$\begin{array}{c} \text{Loss}=W_{1}\cdot L_{1}-W_{2}\cdot L_{2}. \end{array}$$

Then, they are trained on the CIFAR-10 dataset, and the training errors of both networks during the training process are recorded. The experiments demonstrate that it is not the case that the deeper the network depth is, the smaller the training error will be, but instead, the training error will be larger after a long period of training, a phenomenon that Kemin He's team calls network degradation [14]. The residual structure is divided into two parts: the left half is the residual part, which will increase or decrease the convolutional layers according to the network size; the right half is the direct link part. So, the composition of a residual block can be expressed by the following equation:

(2) $$\begin{array}{c} F\left(x_{l+1}\right)=F\left(x_{l}\right)-x_{l}. \end{array}$$

Since in the residual structure, the left and right halves are added by Add (the two feature maps to be added need to have the same number of layers with the same length and width), occasionally the number of layers of the output feature map of the left half is different from that of the right half, which requires adding a point-by-point convolution (the convolution kernel size is 1 × 1) to up-dimension or down-dimension the input in the inline part. The number of channels of the output feature map in the left half and the number of channels of the output feature map in the right half are the same, and Equation (2) is transformed into

(3) $$\begin{array}{c} F\left(x_{l+1}\right)=F\left(x_{l}\right)-h\left(x_{l}\right). \end{array}$$

The right half of the Sensient layer is the densely connected structure of Dense Net, which can speed up the flow of information and improve the information reuse rate; the left half is a directly connected structure with point-by-point convolution, which reduces or increases the dimension of the feature map by point-by-point convolution so that the number of channels of the input transition layer is the same as the number of channels of the input transition layer in the right half [15]. At present, the virtual reality simulation system built in this chapter is mainly built through the Unity3D platform, which mainly realizes related functions such as virtual human driving and virtual pitching. Then, the output feature maps of the two branches are stitched into one feature map in the channel dimension, and finally, the information flow occurs between the channels of the stitched output feature map through the transition layer, and the dimensionality is reduced to decrease the number of parameters.

The input size of the backbone network model constructed in this paper is 416 × 416, and then, the size of the feature map is changed to 208 × 208, 104 × 104, 52 × 52, 26 × 26, and 13 × 13 by downsampling five times in a turn, and the network parameters are changed as shown in Table 1. The downsampling effect can be achieved in the DBL layer by setting the convolution step to 2. In the CSP layer, the downsampling effect is achieved by setting the pooling step to 2 through maximum pooling. In the CSPDN layer, only feature extraction is performed for the feature map, and no downsampling is performed. Also, in the transition layer of CSPDN, only the number of channels is adjusted for the feature map, no downsampling is performed, and the original size output is maintained.

The right branch is used to extract contextual information, and to enlarge the perceptual field, i.e., to increase the mapping area of each pixel in the convolutional output feature map on the input image, the convolutional kernel size of 3 *∗* 3 is used in this branch, which combines the depth-separable convolution with the hole convolution, and its hole rate is 2. The hole convolution injects holes into the convolutional kernel of ordinary convolution, which not only increases the perceptual field but also makes the output features of convolution contain a larger area of information and ensures that the spatial resolution is not reduced. In the semantic segmentation task, increasing the perceptual field can detect and segment larger targets without decreasing the feature resolution, which can make the target localization more accurate.

Although the EBR module uses the null convolution to enlarge the perceptual field, the multiscale context information is not extracted because the null rate does not change. To obtain multiscale contextual information, it is necessary to introduce a multilevel null convolution of the null rate [16]. To reduce the computation and speed up the operation, a one-dimensional factorization of the null convolution is used in the right branch, i.e., the original two-dimensional 3 *∗* 3 null depth-separable convolution is decomposed into two one-dimensional 3 *∗* 1 and 1 *∗* 3 null depth-separable convolutions with a variable magnitude of the null rate for obtaining multiscale contextual information.

(4) $$\begin{array}{c} W^{\prime }=\frac{W+K-2P}{S}-1. \end{array}$$

Therefore, the convolution operation picks different step sizes and fill values to enable downsampling operations on the features. The output features of the first downsampling block enter the new residual block EBR module proposed in this chapter for the convolution operation, where the convolution hole rate of the right branch of the EBR module is 2, without changing the size of the input features and the number of channels.

**Figure 2:**
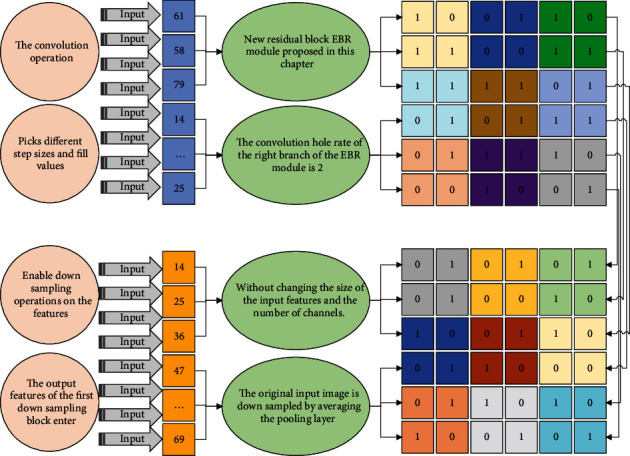
Schematic diagram of channel rearrangement operation.

If the target cannot be detected, it will return to the data receiving module for monitoring. In the overall encoder network, the original input image is downsampled by averaging the pooling layer. Specifically, the original input image is first downsampled by a factor of 2 to obtain an output feature with channel 3 (the original input image is a 3-channel RGB image) and an image resolution of 512 *∗* 256, and this output feature is spliced with the third 3 *∗* 3 convolution feature in the encoder with channel 32 and an image resolution of 512 *∗* 256 to obtain a superimposed feature with channel 35 as the input features of the first downsampling block of the encoder. Then, the original input image is downsampled by 4 times to obtain the output feature with channel number 3 and image resolution 256 *∗* 128, and this output feature is spliced with the feature with channel number 64 and image resolution 256 *∗* 128 obtained from the first downsampling block of the encoder and the feature with channel number 64 and image resolution 256 *∗* 128 obtained from the three EBR modules to obtain the superimposed feature with channel number 131. The superimposed features with the channel number 131 enter the second downsampling module in the encoder, as shown in Figure 2.

The main purpose of the experiment is to study a real-time semantic segmentation method that can be applied to mobile devices, so it is not necessary to use the highest resolution image, and the input image resolution of 512 *∗* 1024 is the result of the original image downsampling [17]. In the final testing phase, the segmentation results of the network model were upsampled to 1024 *∗* 2048 and then uploaded to the Cityscapes website for evaluation. During the training process, as the number of generations increases, the training loss function of the model is decaying and the accuracy gradually improves.

### 3.2. Analysis of Unity3D Interaction Method

The virtual space station system requires that the basic structure of the space station, the composition, structure, function, and structural layout of all the subsystems and products of the environmental control and life protection can be visually displayed in a realistic 3D virtual environment. At the same time, the roaming character should be manipulated to interact with the space station unit to realize the functions of equipment maintenance training, maintenance operation simulation, and visualization maintenance operation guidance. All the above functions are realized based on the construction of a software simulation environment with a high degree of realism. After completing the data calibration, the converted data is fused into the data fusion module and displayed. To ensure the authenticity of the virtual space station user's experience, the original design model is used as the basis for the design of the space station compartment and equipment structure. Based on the model construction, to ensure the fidelity of the display, the model needs to be mapped, lit, and rendered concerning the real equipment data, such as photos, materials, installation environment, and other information, to finally get a virtual reality model with highly realistic visual effects.

The default unit in Unity3D is a meter, so it is also used in 3 ds Max. Set the unit of the model in the Unit Settings tab under the Customize menu, first select the display unit scaling of the model as Metric-Meter, and then set the system unit scaling to 1 unit = 1 meter in the System Unit Settings.

For the models in the subsystem, according to the classification, many models of the same category are duplicated, and the duplicated models can be copied directly [18]. For a duplicated model object, after the baking is finished, no matter how many are copied, except for the model itself, the other consumption and the resources consumed by an object are about the same, so it also achieves the effect of saving resources.

**Figure 3:**
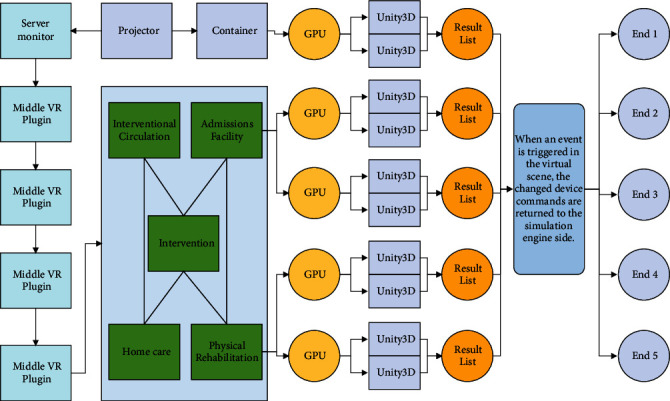
Unity3D architecture.

After all the models are rendered and baked, they need to be exported from the 3 dMax software. The Unity engine supports a variety of file formats for import. The advantage of the FBX format is that it can export the model and its attached lighting and animation and material mapping information together, and it can be recognized in the Unity engine, which greatly facilitates the seamless connection between model making and virtual scenes and avoids the missing information due to the different software environment, as shown in Figure 3.

The simulation engine and virtual scene are two important components of this system. The simulation engine side internally simulates the device state and generates device events and is responsible for distributing these device events to the virtual scene side with messages. The virtual scene side is responsible for the event triggering and animation execution of the objects in the field through the received events. The virtual scene not only provides a visual interface but also supports a variety of interaction methods so that the user can conveniently operate the device [19]. The simulation time can be saved by 12.69% and 13.99% in the case of 250 devices and 500 devices, respectively. When an event is triggered in the virtual scene, the changed device commands are returned to the simulation engine side. The simulation engine side updates the state of the device through the received events and then *M* retreats to the point in time when the child is new to perform simulation calculations. And if a new device control instruction is produced, the device state of the simulation engine side needs to be resimulated in time.

### 3.3. Virtual Reality Real-Time Interactive System Design

To better improve the realism and immersion of the virtual reality simulation system, it is necessary to add an avatar to the VR system as a substitute for a real person in the VR environment. With the continuous development trend of virtual reality technology, avatars have an increasingly important role in VR systems. In terms of virtual human research, virtual human 3D modeling technology and virtual human motion control technology are the most concerned in the industry. The sensors used in environmental perception technology generally include cameras, radars, and GPS. Virtual human 3D modeling technology is already a relatively mature technology, in which the more mature 3D modeling software, such as Autodesk Maya, Cinema 4D, and 3 ds Max, as well as commercial virtual human modeling software, such as Jack and DI-Guy, at the same time virtual human motion control technology also gradually developed. At present, the virtual reality simulation system built in this chapter is mainly built by the Unity3D platform, which mainly implements the functions related to virtual human driving and virtual ball throwing and then studies the consistency of user throwing behavior in the virtual environment based on virtual human driving.

(5) $$\begin{array}{c} V_{i}=\frac{N_{i}\left[n\right]+N_{i}\left[n-1\right]}{\Delta t},\;i=1,2,\ldots ,24. \end{array}$$

**Figure 4:**
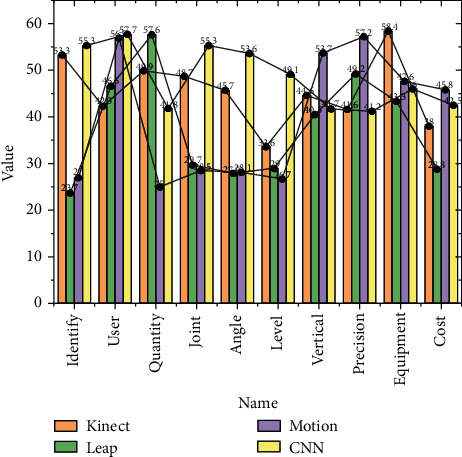
Parameter comparison.

The VR system architecture mainly consists of three parts: the foundation layer, the support layer, and the application layer: the foundation layer provides the underlying hardware support, including Qualisys optical motion capture hardware system, computer server, network communication, and HTC VIVE headset hardware system; the support layer adopts mature commercial software to provide virtual human motion driver, scene simulation, motion data acquisition, etc.; the application layer provides the functions in the corresponding software [20]. The application layer is supported by the corresponding software, through integration and secondary development, to realize the VR scene construction, virtual human loading VR scene, data acquisition, data conversion, and driving virtual human motion and other functions. The cameras collect information around the car by taking pictures and then using convolutional neural networks or traditional image processing algorithms to parse the pictures. The architecture of the motion-capture-based VR system is shown in Figure 4.

Although VR users can visually experience immersive environments, there is not enough research on the consistency of VR users in virtual and real environments. Therefore, it becomes important to explore whether VR users can perform actions in a virtual environment as they do in a real environment. In this section, we investigate the consistency of the throwing behavior in both real and virtual environments. As can be seen from the previous two sections, we developed a virtual reality system in conjunction with optical motion capture technology to drive walking avatars with motion capture data.

(6) $$\begin{array}{c} V_{\text{angle},i}\left[n\right]=\frac{A_{i}\left[n\right]-A_{i}\left[n-1\right]}{\Delta t}. \end{array}$$

The data reception module receives data from each client, and when no target is detected in the data from all clients, the server continuously waits and listens in the data reception module until at least one client detects a target.

The received data is then converted to coordinates in the data calibration module, and if the target is detected to have moved during the process, the module is recalibrated; if, after the module is completed, the target is not detected, it is returned to the data reception module for listening. After the data calibration is completed, the converted data is fused and displayed in the data fusion module. It is difficult for architects or system operation and maintenance personnel to accurately remember the composition and interaction of all resource instances; secondly, the dynamic evolution of the system architecture may introduce some unreliable factors.

## 4. Result Analysis

### 4.1. Analysis of Algorithm Performance Results

To make the training better, the K-means clustering method is used to cluster 5 different sizes of anchor boxes in all data sets. In the subsequent training process, they need to be normalized and then mapped to the output feature map dimensions, and the clustered anchor boxes will be used as the a priori boxes for target detection.

**Figure 5:**
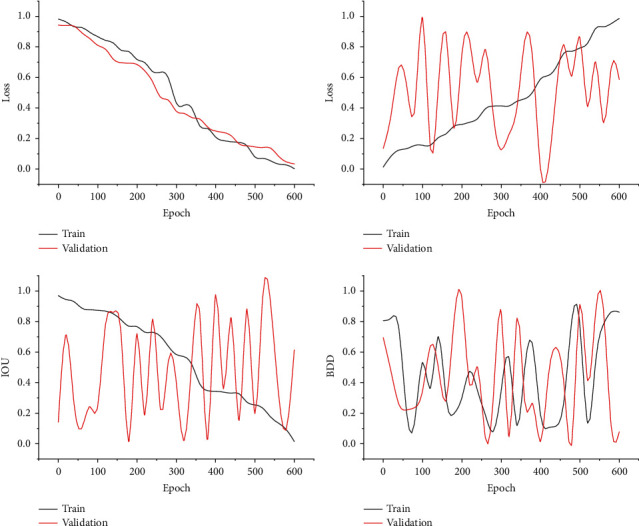
Loss value of target detection branch and AVG IOU value results.

After all the preparations are completed, the network can start training and use TensorFlow's Tensor Board to plot the loss value curve, AVG IOU value curve, and PA value curve during training. The PA value represents the ratio of the number of pixels in the correct prediction category to the total number of pixels in the semantic segmentation. The target detection branch loss graph and AVG IOU value graph are shown in Figure 5.

The training set of the BDD100K dataset is too large, so this paper extracts 3000 images from the training set of BDD100K and then mixes them with the self-picked 3000 training sets to form a new training set. The batch size is 8, and the training set is 6000, so the step of each epoch is 750. The learning rate is initially set to 0.01, and during the training process, the learning rate is initially set to 0.01, and during the training process, if all 4 epochs do not decrease, the learning rate is multiplied by 0.8, e.g., the initial value is 0.01, multiplied by 0.8 to 0.008, and then the training continues. Each layer is followed by a batch normalization layer and an activation layer using the ReLU function.

From the loss curve, we can see that when the epoch is 120, the training loss value decreases very slowly, especially the verification loss value starts to level off. In the AVG IOU curve, when the epoch is 160, the training AVG IOU value increases more slowly, while the validation AVG IOU value starts to decrease slowly. From the AVG IOU curve, we can see that at epoch 180, the AVG IOU is about 88%, which indicates that the training of the target detection branch network has converged and the training can be stopped by saving the weights.

**Figure 6:**
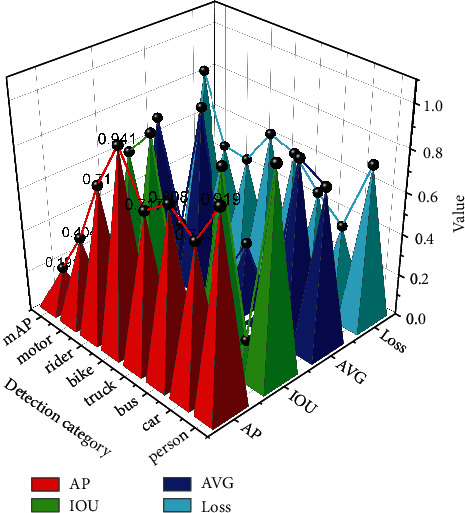
Performance parameters of the multitask convolutional neural network.

1000 images were randomly selected from the BDD100K test set and then mixed with the self-picked 1000 test set images to form a test set with 2000 images. The mAP and more values of the multitask convolutional neural network are tested in the test set, and the test results are shown in Figure 6.

From Figure 6, the mAP of the target detection branch is 67.17% and the mIOU of semantic segmentation is 78.43%. In particular, the AP values of pedestrians and vehicles are 76.72% and 79.11%, respectively, and this accuracy has reached the project requirements. Several mainstream lightweight networks are trained under the same training set and then tested in the same test set, the mAP of the network proposed in this paper is the highest, and the AP values of the network in this paper are the highest in 5 out of 7 categories, which effectively proves that the network in this paper not only has good detection accuracy but also has high generalization ability.

### 4.2. Analysis Results of the Unity3D Virtual Reality Real-Time Interaction System

The system functionality testing is based on the application relationship architecture visualization system to design test cases. Functional testing is performed for each module in this system, and black-box testing is used to test the system login module, visual scene design module, configuration management module, and system management module. The focus is on whether the test results are consistent with user expectations. The following functional tests are conducted for the main functional modules. Security testing is for system users to ensure that user information is not threatened, information theft, and monitoring. The system is tested for user login, role permissions, system data, and interface testing security testing.

**Figure 7:**
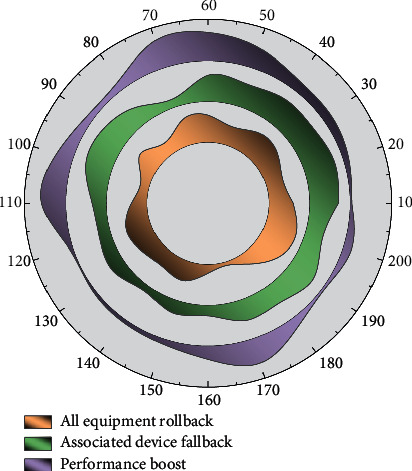
Simulation synchronization runtime.

As the number of devices emulated after the actual deployment in both scenarios and the number of messages manually triggered at the virtual scenario, side is not enough to cause enough pressure on the computing resources of the server, and the emulation engine side can keep backing up in time to process the reported events and again lead the virtual scenario side for a sufficient period. To test the efficiency of network synchronization, a test environment was manually created within the virtual scene with the following conditions: 20 random events were generated within 1 minute (virtual timeline) in the virtual scenario, and the simulation synchronization efficiency of the simulation engine was tested when there were 250 and 500 nodes in the scenario, respectively. The application relational architecture visualization system can help us identify the problems existing in the overall architecture of the enterprise and at the same time assist the enterprise to establish an advanced, fast, and easy-to-use system. The simulation engine is set to simulate 750 s, and the operation mode is set to virtual for the test. Ignoring the effect of network packet loss, the simulation engine can handle all the received events completely and correctly, as shown in Figure 7.

As shown in Figure 7, the simulation time savings are 12.69% and 13.99% for 250 devices and 500 devices, respectively, when comparing the rollback of all device states within the simulation to the rollback of only the relevant devices. In contrast, which supports up to 500 devices in a single host, can fully simulate all the states with the model and correctly obtain all the simulation data, but the inclusion of the fallback mechanism leads to a further reduction in the running speed, which is already lower than the real-world running time, resulting in the virtual scene side running at a speed slower than the real-world time flow, and the acceleration function is also disabled.

**Figure 8:**
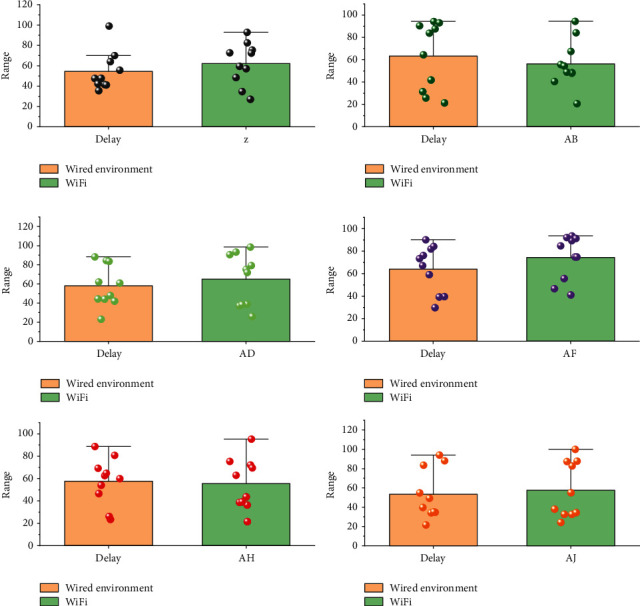
User interaction latency test results in different environments.

To better quantify the latency of the interactive system in this paper to evaluate the real-time performance, this paper evaluates and quantifies the latency of the proposed system from three aspects: computational processing latency, video transmission latency, and user interaction latency. The computational processing latency is the time delay of the HTC Vivi sensor processing human movement signal and the user's computer; according to the official data of HTC, the computational processing part of the latency is about 3 ms. The video transmission delay is about 15 ms. This paper aims to study the real-time semantic segmentation algorithm based on DCNN so that it can perform better on the existing street scene segmentation data set, to provide strong technical support for future industrialization. The user interaction latency is the latency generated by the data transmission through the server during the multiuser interaction. For better quantification, this paper takes user A and user B as an example and calculates the difference between the time when user A receives action data from user B and the time when user B sends action data by adding timestamps to the skeletal data frames of both users and takes the average value as the user interaction delay for multiple tests. In this paper, we test and record the time delay of user and server in wired and wireless environments, respectively, as shown in Figure 8.

From the test results, the total latency of the VR interactive system in the wired connection environment is about 19.28 ms, and the total latency in the Wi-Fi environment is about 30.14 ms. According to the current VR recognized research, when the latency is controlled below 20 ms, no user will have rejection; between 20 ms and 40 ms, there will be a small number of users who are sensitive to latency due to physical reasons. To achieve a more immersive and interactive effect, it is necessary to update the motion capture equipment and design new motion capture methods and solutions. A small number of users who are sensitive to the delay due to their physique will have rejection reactions between 20 ms and 40 ms. As a result, the system implemented in this paper is suitable for any user in a wired environment and still suitable for most users when working in a Wi-Fi environment.

The skeletal data sent includes the ID of the data, because every time the skeletal data is sent, the ID of the skeletal data increases to ensure that the receiver receives the maximum ID information, that is, the latest information about the position of the avatar, and the root position of the avatar and the rotation value of each joint. The joint rotation values need to be further processed, because we first obtain the angle information, and then need to convert the angle information into joint information.

## 5. Conclusion

In this paper, we first introduced the basic theory of convolutional neural networks, including the principles and roles of the components of convolutional networks. Secondly, the data sets used for semantic segmentation and the evaluation criteria for semantic segmentation results are introduced. Finally, two typical real-time semantic segmentation methods based on fully convolutional networks, Fast-SCNN and BiSeNet, are introduced, and their effectiveness in real-time semantic segmentation is experimentally verified, and the advantages and disadvantages of both methods are further analyzed. A virtual interactive system is designed for virtual training scenarios. In this paper, we first investigated the VR systems for virtual training and found that most of the virtual training systems are still in the state of stand-alone offline training. Because of the high resolution of its feature maps, the encoded spatial information is richer. At the same time, many VR applications currently have very limited support for virtual interaction due to the complexity of the interaction and the difficulty of the design of the virtual interaction system. The simulation engine was extended by adding a weather module and a network synchronization module to realize the synchronization function between the virtual scene end and the virtual scene end. Although the two ends run at the same rate most of the time, the simulation engine is always ahead of the virtual scene and simulates the device's behavior in advance. When encountering events reported by the virtual scenario, the strategy of selectively backing off events based on device relevance further improves the simulation efficiency by 12%.

## Figures and Tables

**Table 1:** Variation of parameters in each layer of the backbone network.

Layer name	Input size	Output size
Input	416 × 416 × 3	416 × 416 × 320
DBL	416 × 416 × 3	8 × 208 × 32
DBL	208 × 208 × 32	104 × 104 × 64
CSP	104 × 104 × 64	52 × 52 × 128
CSP	52 × 52 × 128	26 × 26 × 256
CSPDN	26 × 26 × 256	26 × 26 × 256
CSP	26 × 26 × 256	13 × 13 × 512
DBL	13 × 13 × 512	13 × 13 × 512

## Data Availability

The data used to support the findings of this study are available from the author upon request.

## References

[1] Liu X., Sohn Y. H., Park D. W. (2018). Application development with augmented reality technique using Unity 3D and Vuforia[J]. *International Journal of Applied Engineering Research*.

[2] Putra R. S., Utami D. Y. (2018). Pemanfaatan virtual reality pada perancangan game fruit slash berbasis android menggunakan unity 3D[J]. *Jurnal Teknik Komputer*.

[3] Zhang Q., Chang N., Shang K. (2020). Design and exploration of virtual marine ship engine room system based on Unity3D platform. *Journal of Intelligent and Fuzzy Systems*.

[4] Zhu X. (2019). Behavior tree design of intelligent behavior of non-player character (NPC) based on Unity3D. *Journal of Intelligent and Fuzzy Systems*.

[5] Khode A., Kapse P., Besekar R. (2021). College campus virtual experience simulator[J]. *International Journal of Research in Engineering, Science and Management*.

[6] Xie J., Yang Z., Wang X., Wang Y (2018). A remote VR operation system for a fully mechanised coal-mining face using real-time data and collaborative network technology. *Mining Technology*.

[7] Ye F., Li Y. (2021). Research on 3D modeling software Maya digital media animation assisted brain surgery technology. *Journal of Imaging Science and Technology*.

[8] Li S., Ling Y., Yu H., Chen Y. (2014). Research on underwater fish species identification model and real-time identification system. *Smart Agriculture*.

[9] Kokkas A., Vosniakos G.-C. (2019). An Augmented Reality approach to factory layout design embedding operation simulation. *International Journal on Interactive Design and Manufacturing*.

[10] Fachri M., Khumaidi A., Hikmah N. (2020). Performance analysis of navigation ai on commercial game engine: Autodesk stingray and Unity3D[J]. *Jurnal Mantik*.

[11] Yamashita R., Nishio M., Do R. K. G., Togashi K. (2018). Convolutional neural networks: an overview and application in radiology. *Insights into imaging*.

[12] Cong I., Choi S., Lukin M. D. (2019). Quantum convolutional neural networks. *Nature Physics*.

[13] Khan A., Sohail A., Zahoora U., Qureshi A. S. (2020). A survey of the recent architectures of deep convolutional neural networks. *Artificial Intelligence Review*.

[14] Zhou D.-X. (2020). Universality of deep convolutional neural networks. *Applied and Computational Harmonic Analysis*.

[15] Lindsay G. W. (2021). Convolutional neural networks as a model of the visual system: past, present, and future. *Journal of Cognitive Neuroscience*.

[16] Yao P., Wu H., Gao B. (2020). Fully hardware-implemented memristor convolutional neural network. *Nature*.

[17] Dhillon A., Verma G. K. (2020). Convolutional neural network: a review of models, methodologies and applications to object detection. *Progress in Artificial Intelligence*.

[18] Soffer S., Ben-Cohen A., Shimon O., Amitai M. M., Greenspan H., Klang E. (2019). Convolutional neural networks for radiologic images: a radiologist’s guide. *Radiology*.

[19] Kamilaris A., Prenafeta-Boldú F. X. (2018). A review of the use of convolutional neural networks in agriculture. *The Journal of Agricultural Science*.

[20] Riyaz S., Sankhe K., Ioannidis S., Chowdhury K (2018). Deep learning convolutional neural networks for radio identification. *IEEE Communications Magazine*.

